# Simulation, Fabrication and Analysis of Silver Based Ascending Sinusoidal Microchannel (ASMC) for Implant of Varicose Veins

**DOI:** 10.3390/mi8090278

**Published:** 2017-09-14

**Authors:** Muhammad Javaid Afzal, Shahzadi Tayyaba, Muhammad Waseem Ashraf, M. Khalid Hossain, M. Jalal Uddin, Nitin Afzulpurkar

**Affiliations:** 1 Department of Physics, The University of Lahore, Lahore 54000, Pakistan; javaidphy@gmail.com; 2 Department of Computer Engineering, The University of Lahore, Lahore 54000, Pakistan; 3 Department of Physics (Electronics), GC University, Lahore 54000, Pakistan; muhammad.waseem.ashraf@gmail.com; 4 Institute of Electronics, Atomic Energy Research Establishment, Bangladesh Atomic Energy Commission, Dhaka 1349, Bangladesh; khalid.baec@baec.gov.bd; 5 Department of Applied Physics, Electronics and Communication Engineering; Islamic University, Khustia 7003, Bangladesh; mju.aece@gmail.com; 6 Department of Mechanical Engineering Technology (MCET), Higher Colleges of Technology (HCT), Ras al-Khaimah POBox 4793, UAE; afzulpurkar.n@gmail.com

**Keywords:** ANSYS, bioengineered vein, bioMEMS, fuzzy logic, microfluidics, microchannels, varicose vein

## Abstract

Bioengineered veins can benefit humans needing bypass surgery, dialysis, and now, in the treatment of varicose veins. The implant of this vein in varicose veins has significant advantages over the conventional treatment methods. Deep vein thrombosis (DVT), vein patch repair, pulmonary embolus, and tissue-damaging problems can be solved with this implant. Here, the authors have proposed biomedical microdevices as an alternative for varicose veins. MATLAB and ANSYS Fluent have been used for simulations of blood flow for bioengineered veins. The silver based microchannel has been fabricated by using a micromachining process. The dimensions of the silver substrates are 51 mm, 25 mm, and 1.1 mm, in length, width, and depth respectively. The dimensions of microchannels grooved in the substrates are 0.9 mm in width and depth. The boundary conditions for pressure and velocity were considered, from 1.0 kPa to 1.50 kPa, and 0.02 m/s to 0.07 m/s, respectively. These are the actual values of pressure and velocity in varicose veins. The flow rate of 5.843 (0.1 nL/s) and velocity of 5.843 cm/s were determined at Reynolds number 164.88 in experimental testing. The graphs and results from simulations and experiments are in close agreement. These microchannels can be inserted into varicose veins as a replacement to maintain the excellent blood flow in human legs.

## 1. Introduction

**Figure 1:**
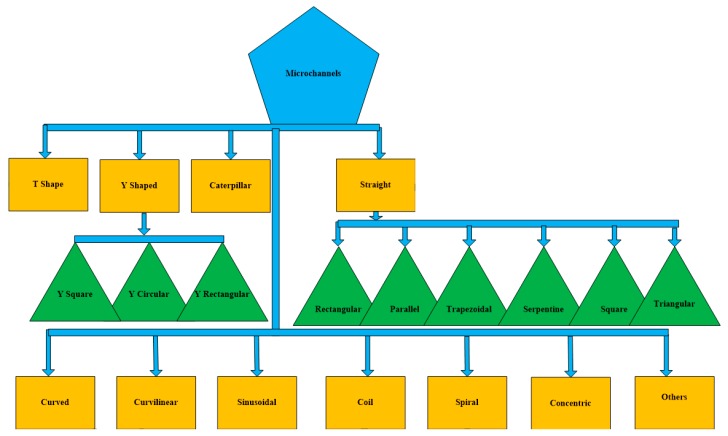
Microchannel classification.

Bioengineered veins give new hope for people lacking healthy and strong veins. There are several main challenges in the implantation of bioengineered veins for medicinal treatments. One of them is that the implanted veins were being rejected by the human body. The humanoid immune system attacks these external objects, perceiving them as harmful invaders. Finally, the first bioengineered vein was implanted successfully, marking a breakthrough in bioengineered humanoid tissues in 2013 [1]. In this decade, bio microelectromechanical system (BioMEMS) techniques have established great potential for use in the field of bioengineered tissues (veins), biotechnology, biomedicine, and microfluidics. They also have a wide range of applications, including drug delivery, medicine, sample preparation, drug screening, blood filtration, blood transport, genetic analysis, cell separation, biochemical analysis, chemical synthesis, and electro-chromatography. In recent times, this technology has the potential of adding new capabilities to the functionality of current surgical devices, which allow surgeons to develop new techniques and procedures [2,3]. Here, the objective is to present the concept of miniaturization in microfluidics with the application of BioMEMS technologies [4]. Microfluidics addresses the performance, precise control, and handling of miniaturized quantities (10^−6^–10^−18^ L) of fluids. Generally, microfluidic systems consist of microchannels, micropumps, microneedles, micro-chambers, micro-sensors, and micro-mixers. Microchannels can be categorized and designed in any alphabetical letter shape. Some are designed with shapes, like straight, square, serpentine, rectangular, curved, curvilinear, coil, trapezoidal, parallel, spiral, and sine wave. They can also be designed in mixed shapes with an alphabetical letter shape. On the basis of design, microchannels can also be fabricated in alphabetical letters of English e.g., T shape and Y shape. A microchannel classification is shown below in Figure 1.

The complete historical development of microchannels was presented by Kandlikar and Grande (2003) [5]. Microchannels are also called micro heat exchangers for the high heat transfer and the removal of heat. The microchannels were used first time by Tuckerman and Pease (1992) in an innovative application of high heat flux removal [6]. Trapezoidal microchannels were utilized by Abed et al. (2014) with nano fluids in order to observe the heat transfer and turbulent flow behavior with numerical analysis [7]. Shojaeian and Kosar (2016) worked on variable thermophysical properties in circular microchannels for the convective transfer of heat. The results indicated that the variations and slip condition considerably affected the characteristics of thermo-fluid [8]. Lee and Mudawar (2016) used the large straight microchannel heat exchangers for flow boiling in space applications. The transfer of heat with fluid flow was examined in the novel sinusoidal microchannels by experimental and numerical modeling [9].

The design of microchannels helps to analyze the fluid flow and mixing of two or more fluids. Many scientists have worked on fluid flow analysis. Straight and serpentine microchannels were used by Afzal and Kim (2014) for the analysis of flow and mixing of non-Newtonian fluids [10]. Microchannels of Polymer Electrolyte Membrane (PEM) fuel cells were used by Ashrafi et al. (2016) for the experimental validation and the numerical simulation of droplet dynamics [11]. Simple T-shaped microchannels were used by Cardiel et al. (2016), in order to analyze the flow behavior and formation of micro-micellar-membranes (μMM) [12]. Chen et al. (2016) examined the maximum value of mass flow rates in ratchet like channels numerically, by the Monte Carlo method [13].

An important and promising use of microchannels is to examine the mass flow rate. The flow rate was generated thermally, due to the direct relation of the mass flow rate and increased temperature. Cubaud et al. (2016) have examined the behavior of threads of viscous fluids, influence of fluid injection schemes, and flow rates experimentally [14]. Law et al. (2014) investigated the increased heat flux flow rate in oblique-finned channels [15]. Law and Lee (2015) examined the pressure instabilities and temperature in straight and oblique-finned microchannels during flow boiling [16]. Law et al. (2016) increased the oblique angles up to 30°–50°, in order to analyze the pressure and heat transfer characteristics in oblique-finned microchannels [17]. Li et al. (2016) worked on curved rectangular microchannels by continuously varying the curvature for novel flow development [18]. Andreussi et al. (2015) worked on the flow regimes in micromixers of T-shaped microchannels by using direct numerical simulations [19]. Li et al. (2016) used curved microchannels and measured the elasticity-induced unstable viscoelastic flow structures [20].

The sinusoidal design and the miniaturized form of the microchannels have been used to separate the small particles from larger particles successfully. Liu et al. (2015) used straight rectangular microchannels for the sheathless separation of bacteria and red blood cells, by using viscoelastic effect technique [21]. Liu et al. (2015) worked on the size based separation of the bacteria from red blood cells (RBCs) in different straight microchannels by using viscoelastic effects technique [22]. Rafeie et al. (2016) worked on a multiplexing technique in order to build a high throughput system for spiral channels. The technique was used for the ultra-fast blood plasma separation [23]. Nivedita and Papautsky (2013) worked on the continuous separation of blood cells in spiral microchannels with high efficiency [24].

Many other scientists worked on other research parameters of microchannels. Lau et al. (2016) have demonstrated a solution for high throughput of fluids at micron level on the basis of flow cytometry. The existing demonstrations of optofluidic time-stretch imaging were based on the inertial flow microfluidic platforms [25,26,27,28,29,30,31]. Li et al. (2016) have used the cross shaped microchannels in order to study the electrokinetic instability numerically [32]. Liu et al. (2014) have worked on multiplex focusing of particles by using rectangular microchannels with different geometries and Reynolds numbers [33]. Nivedita et al. (2013) worked on Dean Flow for cell focusing at higher flow rates. Their work lead to high efficiency separations at ultra-high throughputs in spiral microchannels [34]. Ma et al. (2014) used rectangular microchannels and studied the three-dimensional (3D) complex nature of the inside flow of droplets. They demonstrated the ability to control the droplet flow environment by adjusting the viscosity ratio between the two phases [35]. Xue et al. (2016) worked on double spiral microchannels for the lateral migration of series of dual droplets by numerical simulation [36]. Zhang et al. (2016) studied the comparison of flow boiling performance in a net of interconnected microchannels [37].

The sinusoidal design of microchannels has better capabilities for cell sorting, cell focusing, fluid flow and heat transfer. Tayyaba et al. (2013) simulated the sinusoidal microchannel for cell sorting systems [38]. Sinusoidal microchannels have a clinical application in circulating tumor cells CTC with a high aspect ratio [39]. Chiam et al. (2016) worked on sinusoidal microchannels for the analysis of heat transfer and fluid flow. The addition of secondary branches, especially in sinusoidal microchannels, led to further enhancement of the heat transfer characteristics. There is one disadvantage, that there is low pressure drop in these channels [40]. Solehati et al. (2014) investigated the mixing performance in T-junction sinusoidal microchannels numerically, at higher Reynolds number [41]. Aliabadi et al. (2016) worked on experimental investigations on the cooling performance of sinusoidal channels [42]. Alshare et al. (2016) analyzed heat transfer and gaseous flow in sinusoidal microchannels with the help of computational modeling [43]. Many researchers have performed investigation on microneedles, micropumps, microchambers, microsensors, and micromixers, for biomedical and other applications [44,45,46,47,48,49,50,51].

**Figure 2:**
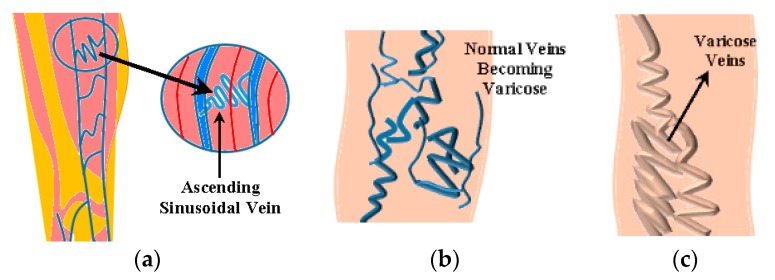
(**a**) Tortuous veins; (**b**) normal ascending sinusoidal veins which becomes varicose veins; and (**c**) swelled varicose veins.

There is widespread use of microchannels in bioengineering technology. The microchannels can also be designed like the structure of veins and arteries. These microchannels are used as bioengineered blood vessels. These vessels are also the integral part and an important innovation in the field of tissue engineering and biomedical engineering. Bioengineered vein implants have proven to be a landmark in tissue engineering. All the veins, including tortuous veins, carry blood from the heart to the other parts of the body and back. There are one-way valves in veins. The valves prevent backward blood flow. The major function of the valve is to make the flow consistent in one direction. Due to the failure of valves and the inconsistency of flow, the blood is collected in the veins and overfills them. Therefore, the veins become dilated, swelled, and enlarged. Often it brings pain, heaviness, aches, bluish–purple or red color, discoloration, ulcers, sores, blood clots, and chronic inflammation in human legs [52]. These tortuous veins could be ruptured in severe cases. These veins are far away from the heart. Hence, gravity makes it harder for the blood to flow upward. Now these tortuous veins are called varicose veins and usually found in legs. The causes of appearance of varicose veins are: the chronic heart valve conditions, pregnancy, menopause, standing for longer periods of time, obesity, and pressure on the abdomen. As a consequence, there is an increase of the blood pressure in the legs. The structure of the varicose veins is different in various body parts. They can be straight, curved, and sinusoidal (ascending and descending) in shape. A portion of such ascending sinusoidal vein is considered for this novel research work. These varicose veins are shown in the Figure 2.

The conventional treatment of such diseases is ligation and stripping. Today, there are also minimally invasive methods as an alternative of surgery, such as endovenous ablation with laser or radiofrequency, and foam sclerotherapy. These treatments may cause bruising and swelling [53]. To overcome these issues, there is a proposed plan to place the silver based fabricated ascending sinusoidal microchannel inside the varicose (tortuous) vein, like a stent. Consequently, the required blood flow can be made possible through these veins. These veins can be fabricated in any shape, design, diameter, and length. They can be sinusoidal (ascending or descending), curved, spiral, and straight. Moreover, the size and shape of the original vein is exactly like the ascending sinusoidal microchannel (bioengineered vein) used in this research. This method can be an alternative of ligation and stripping procedures.

### Complications of Varicose Veins after Ligation and Stripping

There are many complications and risks of varicose vein surgery, such as listed below.

1. Wound complications
2. Leakage of lymph from the groin
3. Deep vein thrombosis (DVT)
4. Pulmonary embolus
5. Vascular injury
6. Damaged femoral vein
7. Vein patch repair

These problems often occur after or during surgery. The issues often arise in lymphatic system after ligation and stripping. The lymphatic system is a part of the circulatory and immune system. This system is disturbed because of chylous leakage, due to surgical procedures in lymphatic system. After surgery, the blood clots are formed in the vein, which are called DVT, usually occurring in the legs. This clot can cause pulmonary embolus and vascular injury. Patient cannot walk with the damage of femoral vein patch after surgery. There are also other treatments like sclerotherapy, radiofrequency treatment, and endovenous laser treatment, but they are very costly. In radiofrequency treatment, the electric signals are produced in the varicose vein to reduce the pain, and the problem may persist even after this treatment. Therefore, it is not considered a successful treatment. The costly laser treatment is also used for stripping veins [54,55,56,57,58,59]. In sclerotherapy, the chemical sclerosant is inserted into a vein for its complete elimination. This destroys the vessel from inside. This results in the formation of a clot which can block blood flow in the vein. Now the blood has to flow from the smaller veins associated with the saphenous vein [60]. The blood pressure in these smaller veins is low. These smaller veins are called tortuous veins. The blood is pumped forward by heart contractions. Hence, pressure in tortuous veins become larger. Therefore, smaller tortuous veins, that feed these varicose veins, can be injured underneath the skin, and due to this issue, the tissue can be damaged [61]. In this study, there is a novel solution for this problem; to use a stent, also called the bioengineered vein in these tortuous veins. This bioengineered vein should be separated from our fabricated silver substrate before use. In this research, the simulation of bioengineered veins has been performed before the actual fabrication, testing, and implantation. For simulation, MATLAB (2013A, Mathworks, Natick, MA, USA) and ANSYS Fluent (ANSYS 17, perpetual license purchased by Ibadat Education Trust, The University of Lahore, Pakistan) have been used. The boundary conditions for simulations are set with the values of the actual blood pressure and velocity in these veins. In tortuous veins, the blood pressure and velocity are normally taken as 1 kPa to 1.5 kPa, and 2 cm/s to 7 cm/s, respectively [62]. In order to verify the same blood flow rate, blood velocity and pressure in these tortuous veins, MATLAB and ANSYS simulations have been performed by considering silver based biomedical microdevices (microchannels). Microchannel characteristics, like design/shape, length, type, flow rate, fluid used, fabrication technique, and applications, have been presented in Table 1. In the next sections, FUZZY and ANSYS based parametric estimation simulations have been done extensively.

## 2. Fuzzy Rule Based Parametric Estimation

In this study, fuzzy logic control (FLC) has been used to estimate the flow rate and velocity of an ascending sinusoidal microchannel. The system has four inputs and two outputs. The inputs are taken as Reynolds number, pressure, curve height, and % loss. Here, % loss means friction and surface roughness. The outputs are taken as flow rate and velocity. Three membership functions are taken for each parameter of inputs and outputs in this microchannel system. The ranges of membership functions are presented below with each parameter of inputs and outputs for ascending sinusoidal microchannel system in Table 2.

In FLC, rule tables are needed to describe the parameters (three inputs and two outputs) with three membership functions [66]. When fluid flows through the microchannel, a considerable amount of frictional force acts upon it and affects the fluid flow. The friction factors for the sinusoidal microchannel were almost constant in the transition region, and gradually increased in the steady flow region.

The Hagen–Poiseuille equation is used for each and every rule of flow rate and velocity [67]. This equation can be used to measure the laminar flow rate through the ascending sinusoidal microchannel.

(1) $$\;Q\;=\frac{\pi R^{4}\;\left(\Delta P\right)}{8\eta L}\;$$

Here, Q represents the fluid flow rate, R represents internal diameter of the microchannel, ΔP represents pressure variation, η represent the fluid viscosity, and L represents the length of the microchannel.

**Figure 3:**
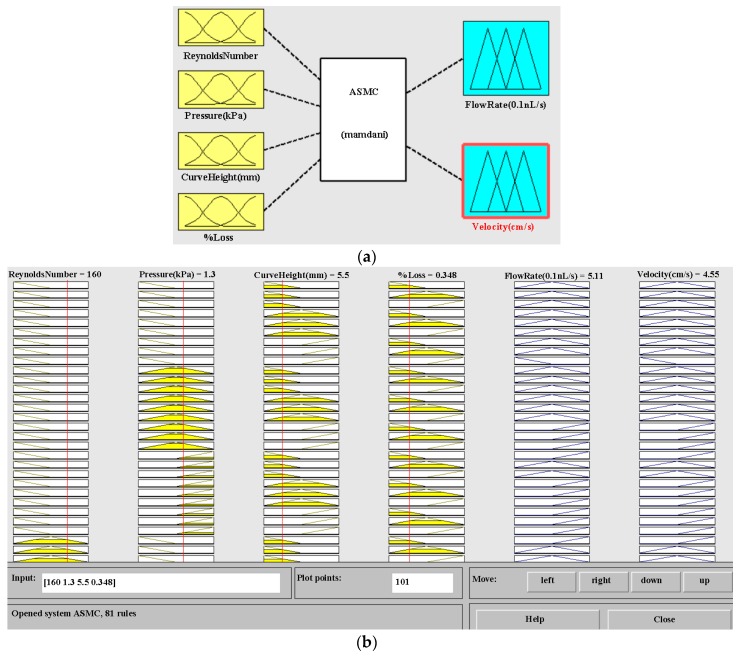
(**a**) Ascending sinusoidal microchannel (ASMC) by using fuzzy logic based inference system editor; (**b**) MATLAB Rule Viewer.

The number of inputs are four, and each input has three membership functions. The formula for the number of rules is membership functions to the power of inputs (3^4^). Therefore, 81 rules are prepared by MATLAB rule editor and viewer with the help of IF-AND-THEN logic [68]. The ascending sinusoidal microchannel system (ASMC) is described, along with MATLAB rule viewer for FLC in Figure 3.

**Figure 4:**
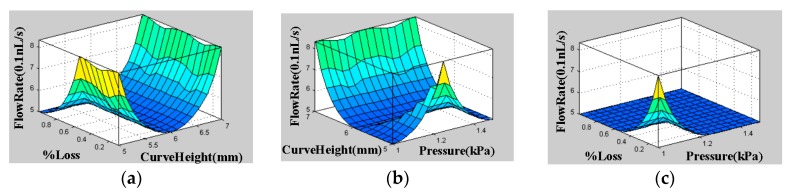

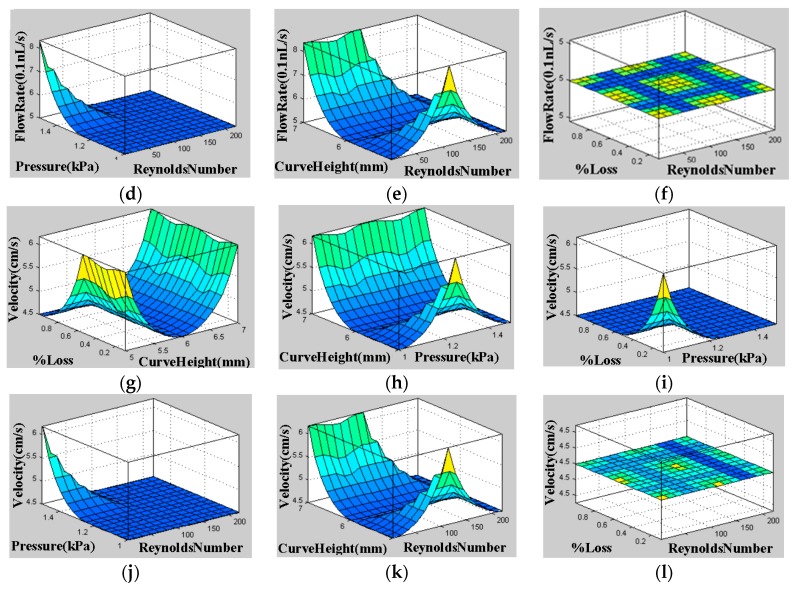
The 3D surface viewer graphs. (**a**) The dependency of flow rate on % loss and curve height. (**b**) The dependency of flow rate on pressure and curve height. (**c**) The dependency of flow rate on % loss and pressure. (**d**) The dependency of flow rate on Reynolds number and pressure. (**e**) The dependency of flow rate on curve height and Reynolds number. (**f**) The dependency of flow rate on % loss and Reynolds number. Figure 4g presents the dependency of velocity on % loss and curve height. (**h**) The dependency of flow rate on pressure and curve height. (**i**) The dependency of velocity on % loss and pressure. (**j**) The dependency of velocity on Reynolds number and pressure. (**k**) The dependency of velocity on curve height and Reynolds number. (**l**) The dependency of velocity on % loss and Reynolds number.

The 3D graphs of surface viewer for curve radius, pressure, % loss with flow rate and velocity, are presented Figure 4. Figure 4a presents the dependency of flow rate on % loss and curve height. Figure 4b presents the dependency of flow rate on pressure and curve height. Figure 4c presents the dependency of flow rate on % loss and pressure. Figure 4d presents the dependency of flow rate on Reynolds number and pressure. Figure 4e presents the dependency of flow rate on curve height and Reynolds number. Figure 4f presents the dependency of flow rate on % loss and Reynolds number. Figure 4g presents the dependency of velocity on % loss and curve height. Figure 4h presents the dependency of flow rate on pressure and curve height. Figure 4i presents the dependency of velocity on % loss and pressure. Figure 4j presents the dependency of velocity on Reynolds number and pressure. Figure 4k presents the dependency of velocity on curve height and Reynolds number. Figure 4l presents the dependency of velocity on % loss and Reynolds number. These dependencies are shown below in Figure 4.

The membership functions have been formulated from *f*_1_ to *f*_6_. After that, Mamdani’s principle is implemented, and the results have been tabulated [69]. The flow rate and velocity comparison, and the error percentage from the values of both models, are given in the following Table 3.

The results of fuzzy rule based parametric estimation are shown in the above table. By applying Mamdani’s principle, the values of flow rate and obtained velocity are 5.15 (0.1 nL/s) and 4.65 (cm/s), respectively. MATLAB simulated values for flow rate and obtained velocity are 5.11 (0.1 nL/s) and 4.55 (cm/s) respectively. The minor error percentages in the values for flow rate and obtained velocity are 0.78% and 2.19%, respectively. The results are in close agreement. Now in the next section, ANSYS Fluent simulation has been presented to verify the MATLAB results for the ascending sinusoidal microchannel system.

## 3. ANSYS Simulation Based Parametric Estimation for ASMC

After the successful simulation of MATLAB FUZZY LOGIC with Mamdani’s model, the computational fluid dynamics (CFD) simulation has been performed for the ascending sinusoidal microchannel. Here, the blood is taken as the non-Newtonian flowing fluid. The flow rate and blood velocity for the ASMC have been determined. The commercial CFD software ANSYS Fluent has been used for simulation. For velocity parameter, different values like 0.02 m/s, 0.03 m/s, 0.04 m/s, 0.05 m/s, 0.06 m/s, and 0.07 m/s, have been used. While, for pressure difference, values like 1 kPa, 1.1 kPa, 1.2 kPa, 1.3 kPa, 1.4 kPa, and 1.5 kPa, have been used [70]. These are the actual values of velocity and pressure in varicose veins. The results of the simulations will be compared with the experiment in the next sections.

The computational domain of ASMC is represented in three dimensions (3D). The channel has been designed with three ascending curves of increasing radii, by using design modeler tool. The length of the channel has been taken as 51 mm. The model is sinusoidal in shape, with the dimension of 0.9 mm for width and the same for depth. The model consisted of inlet, outlet, and the wall. For accurate results, mesh independence has been carried out by varying the cell density of the model for similar boundary conditions. This procedure has been followed for fine mesh tetrahedrons to discretize the computational domain. The meshing has been done for up to 7524 elements and 2541 nodes. Moreover, a high-resolution scheme has been selected as the solving option. The model has been set for two domains, like fluid and solid. In the fluid domain, blood has been used as fluid. Besides, silver has been used in the solid domain. Here, flow regime has been applied at the inlet of microchannels, with normal velocities of 0.02 m/s, 0.03 m/s, 0.04 m/s, 0.05 m/s, 0.06 m/s, and 0.07 m/s. In the set up section of ANSYS Fluent, turbulent kinetic energy and turbulent dissipated rate have been set at 5 m^2^/s^2^ and 4 m^2^/s^3^. In the set up section, the fluid temperature has been taken as 320 K. In calculations, the Reynolds number for the microchannel was determined by the following equation:

(2) $$R_{e}=\frac{\rho D_{h}U_{in}}{\mu }$$

where $R_{e}$ is Reynolds number, $U_{in}$ is velocity in microchannel; μ is viscosity, $D_{h}$ is hydraulic diameter, and ρ is density of the fluid. The Dean number was determined with the following equation.

(3) De=Red2r 

In this equation, *d* is diameter and *r* is the curvature radius of the microchannel. K-epsilon viscous model has been considered in the set up tool of Fluent. Amongst all models at the back end of the ANSYS, the K-epsilon model has been found accurate in predicting the fluid flow [71,72,73,74,75,76]. After selecting the k-epsilon model, power law is selected from the Create/Edit Materials because of the blood rheological property [77]. For the boundary conditions and reference values, the material properties of blood are given to the setup section of ANSYS [78]. The flow rate is determined by the equation: Q=AV. Here, Q is the flow rate, A is the area of cross-section of the microchannel, which is constant throughout, and V is the velocity of the fluid [79].

**Figure 5:**
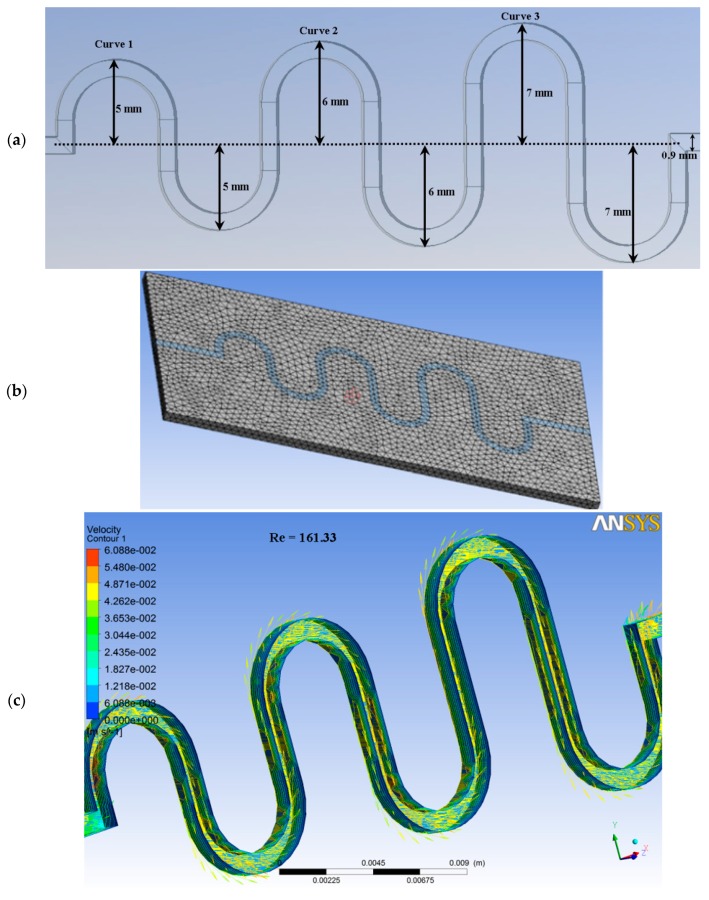

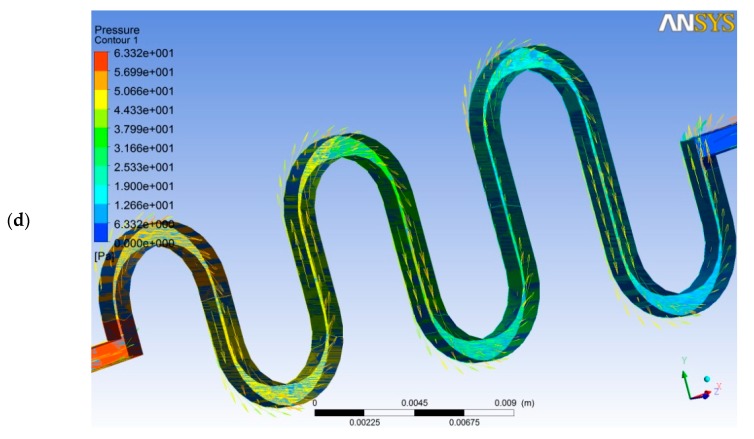
(**a**) Dimensions of microchannels; (**b**) mesh analysis; (**c**) contour plot for velocity; (**d**) contour plot for pressure across the microchannel.

The contour plot illustrates different values of velocity and pressure inside the microchannel. Figure 5 shows the contour plots for velocity and pressure at 1.0 kPa, 1.10 kPa, 1.20 kPa, 1.30 kPa, 1.40 kPa, and 1.50 kPa across the microchannel. The figure below shows that velocity is increased across ASMC, from inlet to outlet. Since the inlet velocity has been set at a low value, the fluid flow is increased along the ascending sinusoidal microchannels. However, the velocity has been changed, due to the changes in the values of pressure along the ascending sinusoidal microchannel. Figure 5 shows the dimensions of microchannels, mesh analysis model and the contour plots for velocity and pressure. The contour plots also show that pressure is decreased from inlet to outlet. Since the inlet pressure was set at a high value, the fluid flow has been decreased along the ascending sinusoidal microchannels. However, the pressure is changed due to the variation in the values of velocity along the ascending sinusoidal microchannel, as shown in Figure 5.

**Figure 6:**
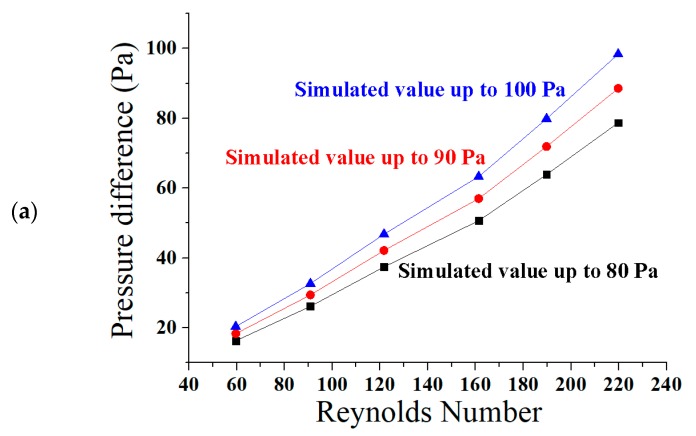

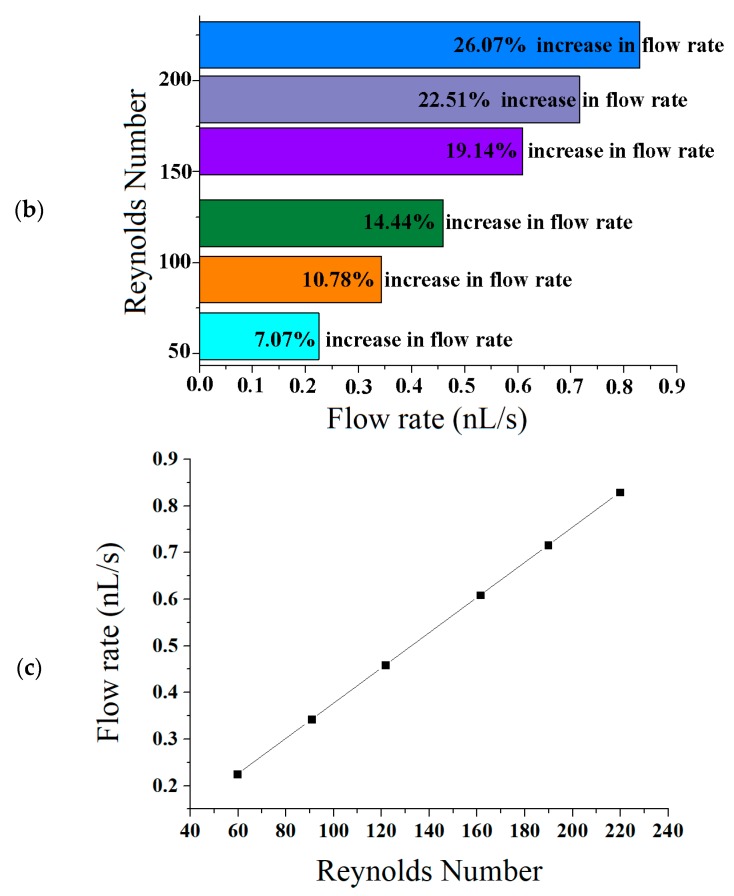
(**a**) Pressure difference vs Reynolds number; (**b**) bar graph between % increase in flow rate and Reynolds number; (**c**) graph between flow rate and Reynolds number across the ascending sinusoidal microchannels.

The pressure is generally increased at the inlet of the channel, and decreased at the outlet. It is clear from the above figure that the pressure drop increases due to an increase in the Reynolds number. It is noted that the higher Reynolds number is actually affecting the pressure drop. The high-pressure fluid pushed the fluid to flow along the microchannel and come out from the outlet region. When the fluid with constant properties flows through the channel, a linear relationship is expected between pressure drop and Reynolds number. However, there are three ascending curves used in this research work. So, when the fluid passes through all the curves, the graph shows a small rise in pressure drop. The simulation has been carried out to identify flow rate, pressure drop, and velocity in the ascending sinusoidal microchannel. Figure 6 shows the pressure drop across microchannels with different Reynolds numbers.

In the above Figure 6a presents that the pressures drop is increased with Reynolds number. The obtained curves are similar to the research work of Chiam et al. (2016) for sinusoidal microchannels [40]. The viscous forces are dominant, therefore, the flow is laminar, as the Reynolds number has been kept less than 220. Figure 6b shows the bar graph between % increase in flow rate and the Reynolds number. The flow rate is increased from 7.07 to 26.07%, while the Reynolds number is increased from 59.57 to 219.76 in the ANSYS simulation. Figure 6c shows that flow rate has a linear relation with Reynolds number. As the Reynolds number increases, the flow rate is also increased. At Reynolds number 219.76, the flow rate of 8.0 (0.1 nL/s) has been obtained. At Reynolds number 161.33, the velocity of 6.088 cm/s and the flow rate of 6.0 (0.1 nL/s) have been achieved. In this novel study, the results of MATLAB FUZZY and ANSYS simulations are found to be in close agreement.

## 4. Fabrication, Testing, and Implant Procedure of ASMC

**Figure 7:**
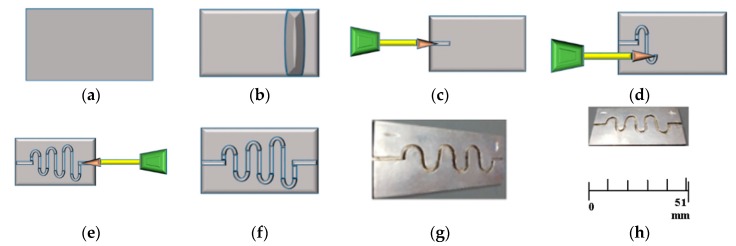
(**a**–**h**) Schematic and actual fabrication by micromachining.

Micromachining is the most diverse category of manufacturing processes available for the fabrication of parts with dimensions of less than 1 mm. Machining is a process in which a cutting tool is used to remove small chips of material from the work piece [4]. First of all, a sheet of silver (51 mm in length, 25 mm in width, and 1.1 mm in diameter) is prepared and ultra-cleaned. With the help of a cutting tool, the small chips of the material are removed from the silver substrate. For this purpose, a relative motion is achieved in this simple micromachining operation by means of a primary motion, called “cutting speed”, and a secondary motion called “feed”. The shape of the tool and its penetration into the work surface, combined with these motions, produce the desired shape of the resulting work surface. Consequently, an ascending sinusoidal microchannel is fabricated with 0.9 mm width and depth, as shown in Figure 7.

Figure 7a presents the substrate of silver, Figure 7b presents the surface smoothing and electro polishing of silver substrate, Figure 7c–e presents the continuity of simple micromachining process, Figure 7f shows the prepared silver substrate with ASMC, Figure 7g presents the digital camera image of the side view of the actual fabrication, and Figure 7h presents the digital camera image of the top view of the actual fabrication process. After the fabrication, the experiment was performed for the verification of results of ANSYS Fluent.

**Figure 8:**
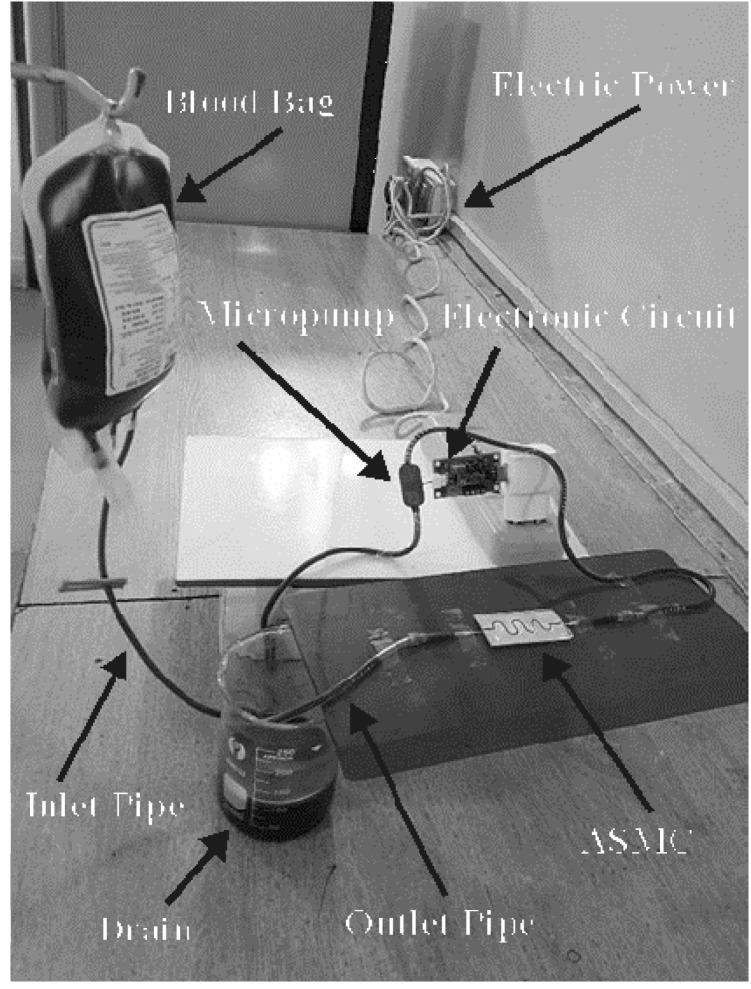
Experimental setup.

The fabricated ascending sinusoidal microchannel was tested for its flow rate and velocity. Actual values of pressures and velocities were used as in ANSYS Fluent simulation. MP 6 micropump was used along with the other experimental apparatus. Pure blood could clot in the MP 6 micropump. Therefore, an anticoagulant heparin was used and mixed in the blood as a blood thinner. It prevented blood clots forming in the MP 6 micropump. MP6 could measure flow rate up to 0–7 mL/min, and could apply pressure up to 550 mbar (55,000 Pa) pressure. There were ports on the microcontroller through which the desired pressure could be made. The microchannel was covered tightly with the glass sheet with glass glue, to avoid the escape of blood out of the channel. Flow rate and velocity were determined at the output. The experimentally obtained values are in agreement with the simulated values. The testing apparatus is shown in Figure 8.

The bioengineered strategies and plans are made before the implantation. Before the implant, the selected area would be shaved, washed, cleaned, and anesthetized. The ASMC (bioengineered vein) will be separated from the silver substrate and implanted into the vein through a thin tube fabricated from medical grade materials, called a catheter. The surgery is similar to an angioplasty technique. In this procedure, blood thinner medication should be used before surgery to diminish the possibility of blood clots. The person will lie straight on an operation table during the procedure. Once the patient is on the implant procedure, a cut is made near the varicose veins. The surgeon then implants the bioengineered vein. The electronic devices will monitor the blood pressure and the heart rate. Satisfactory flow of blood will be restored after the implant, if the surgeon is satisfied with the implant.

## 5. Results and Discussion

In the MATLAB FUZZY LOGIC simulation, the flow rate and velocity were found to be 5.11 (0.1 nL/s) and 4.55 (cm/s), at Reynolds number 160, respectively. In the ANSYS Fluent simulation, the flow rate and velocity were found to be 6.0 (0.1 nL/s) and 6.088 (cm/s), at Reynolds number 161.33, respectively. The results of two simulations were found to be in close agreement. MATLAB is a parametric estimation tool, and it performs a significant role in describing the behavior of the system through mathematical models, while ANSYS Fluent is the most general purpose powerful computational fluid dynamics (CFD) software package. Fluent has extensive physical modeling capabilities, through which the fluid flow can be modeled precisely for microchannels. The discussion has been presented in the following four easy steps.

### 5.1. Reynolds Number, Pressure Difference, and Flow Rate

**Figure 9:**
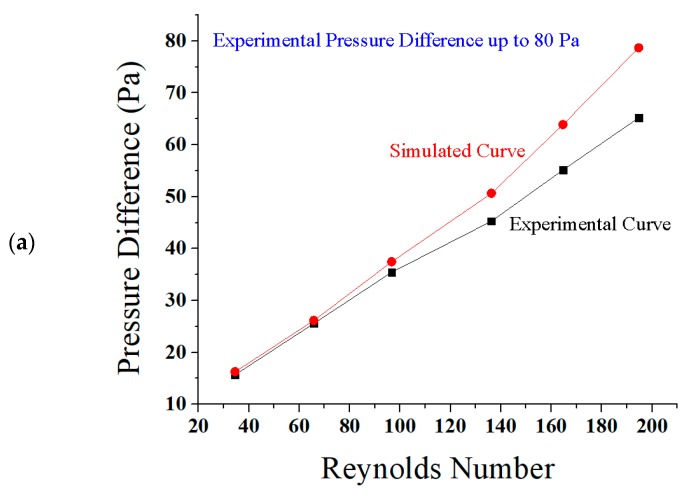

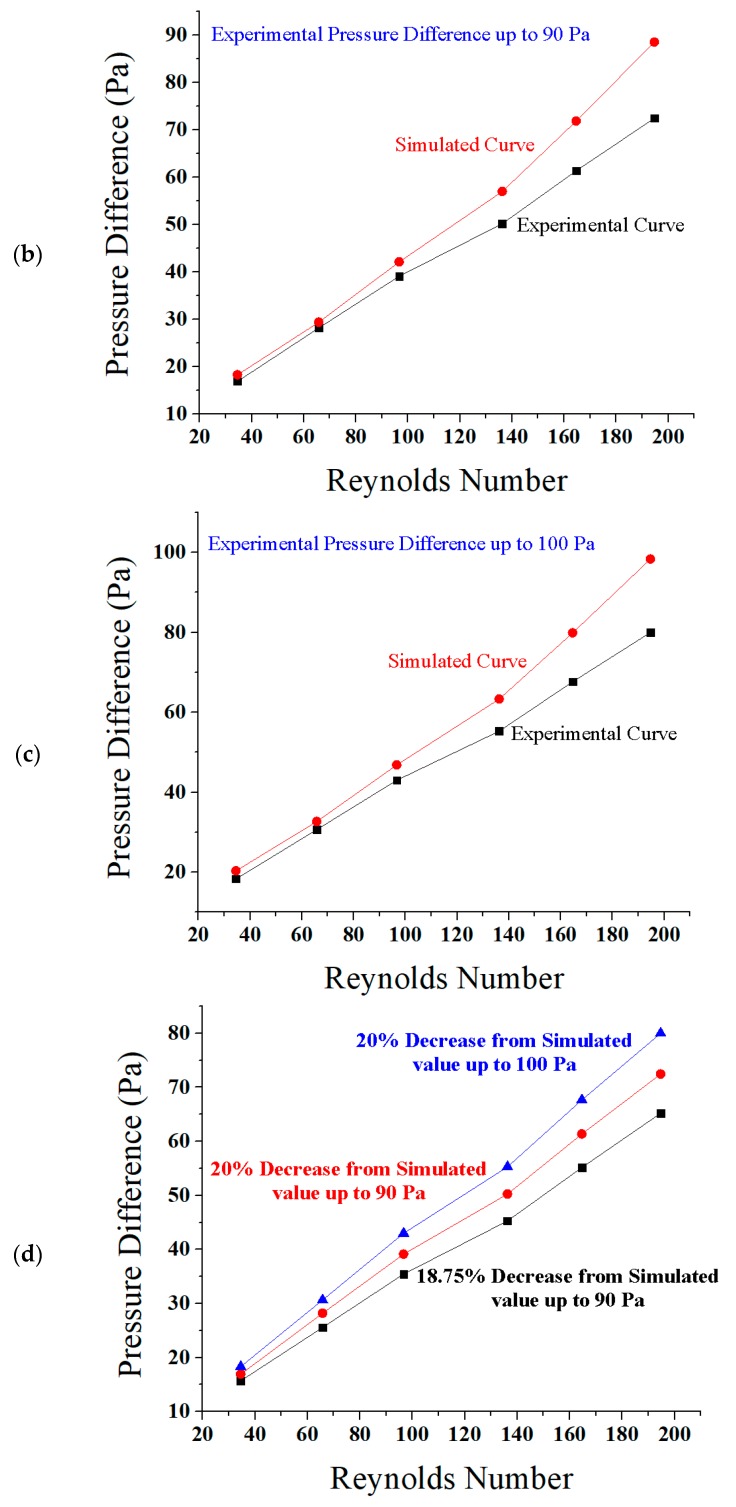

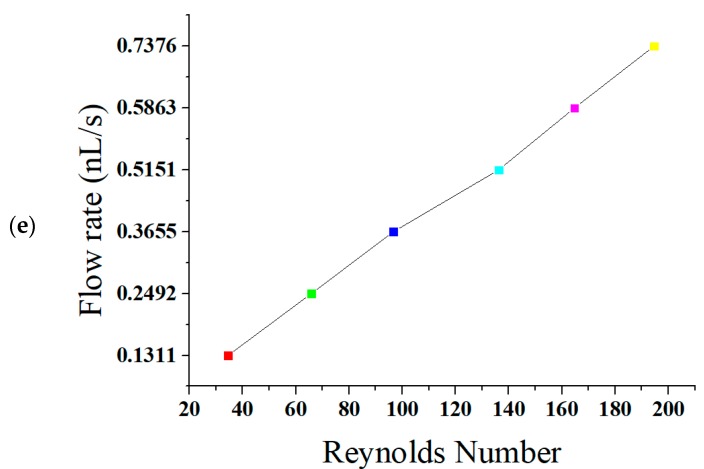
Comparison of graphs between Reynolds number and pressure difference in simulations and experiments (**a**) at 80 Pa; (**b**) at 90 Pa; (**c**) at 100 Pa; (**d**) percentage decrease from simulated data to experimental data; and (**e**) Reynolds number vs. flow rate in testing.

The direct relation of Reynolds number and the pressure difference is discussed here with some experimental deviations. The reasons for these deviations were the frictional factor in the path of the fluid, and the ascending nature of the microchannel [80]. The frictional factor was set as turbulent kinetic energy, and dissipation rate in the setup tool of ANSYS Fluent [81]. The graphs shown in Figure 9a–e are between Reynolds number and pressure difference.

In Figure 9a,b both curves are almost matched at Reynolds numbers less than 100. The graphs have shown a little deviation for Reynolds numbers greater than 100. It has been observed that the flow is laminar throughout the channel for all curves. The slope of the graphs based on simulation is more increased than the curves based on experiments for the Reynolds number 100. Figure 9c presents a collective comparison of decrease in the values of pressure difference in experimental testing. There is a decrease in the values of pressure difference in the experiment as 20% at 100 Pa, 20% at 90 Pa, and 18.75% at 80 Pa. The reason for this deviation is the frictional factor and the ascending nature of the microchannel, as explained above. The direct relation between Reynolds number and the pressure difference is shown below in the graph Figure 9d. The curves are almost matched at all Reynolds numbers. The flow is laminar throughout the ascending sinusoidal microchannel for all simulated and experimental curves. There is also a similar decrease found in the values of pressure difference in experimental testing from the simulated data. The direct relation of Reynolds number and the experimental flow rate is discussed in the next graph in Figure 9e. This experimental graph presents a little deviation because of frictional factors and the ascending nature of the microchannels, as discussed above. Flow rate is increased gradually along with the Reynolds number. At Reynolds number 164.88, the flow rate and velocity are found as 0.5843 nL/s or 5.843 (0.1 nL/s), and 0.05843 m/s or 5.843 cm/s, respectively.

The Dean number and the corresponding Reynolds number has been calculated for all three curves of ASMC. For the first 5 mm curve of ASMC, the Dean numbers were 10.347, 19.737, 29.01, 40.875, 49.407, and 58.404. For the second 6 mm curve of ASMC, the Dean numbers were 9.3123, 17.7633, 26.109, 36.7875, 44.4663, and 52.5636. For the third 7 mm curve of ASMC, the Dean numbers were 8.6225, 16.4475, 24.175, 34.6625, 41.1725, and 48.67. This data clearly shows that the Dean number and Reynolds number are in direct relation. It also shows that the Dean number decreases with the increase of radius of curvature. From Equations (2) and (3), it is clear that the Dean number has an inverse relation with viscosity of fluid. Therefore, the Dean vortices are not very prominent.

### 5.2. Implantation of Bioengineered Veins and Related Issues

The biomedical application of the fabricated and tested ASMC is the implantation of bioengineered veins in varicose (tortuous) veins. All surgical problems can lead to a main problem of stopping the blood flow through the great saphenous vein. Actually, this ligation stops the blood flow through this passage now. There are other natural smaller tortuous veins in legs for blood flow. If blood flows from these smaller tortuous veins, then quantity of flowing blood becomes large. Therefore, pressure of blood can be disturbed in these tortuous veins. Persistence of pressure to the femoral nerve and in these tortuous veins can prevent blood from flowing. The decreased or increased blood flow can result in damaging of tissues. The recovery from varicose vein surgery is also very slow and painful. The stripping and ligation of great saphenous vein can also cause problems in doing extensive exercise [59]. The blood should pass through the same veins as it was flowing before ligation. Therefore, implant of bioengineered veins is proposed for both the varicose (tortuous) and saphenous veins. For this purpose, ASMC has to be separated as bioengineered vein from the silver substrate. The proposed option for the cure of this disease and to make blood flow better is to use a bioengineered vein (silver microchannel) in the varicose vein. There are similar examples of stent implantation [82,83,84,85]. The bioengineered blood vessel also appears to be safe for dialysis process.

There is an urgent need for implantation of such biomedical devices in order to improve the current biomedical condition of old age patients. The majority of these bioengineered veins are made of metal and metal alloys. The corrosion issue was the first to be reported. The concerns about corrosion implications from these implants are minor as compared to the relief that patients feel and get from the anticipated beneficial effects [86]. Silver metal is considered in this novel work because of its low cost and biocompatibility. Few examples already exist of using silver implant in human body. A silver medical device can also be implanted into the vascular system, esophagus, trachea, colon, biliary tract, and urinary tract [87,88]. Our artificial silver bioengineered veins (microchannels) cannot dilate, swell, and enlarge. Therefore, no pain, heaviness, aches, bluish–purple or red color, discoloration and ulcers, sores, blood clots and chronic inflammation occurs in legs. Supply of blood can be maintained properly in them. Elasticity is another issue; there is no elasticity like natural veins in this artificial new vein. In old age, the tortuous veins shrink because of reduced elasticity. Therefore, these types of veins can be suitable for inflating the blood vein with better blood flow. As these veins are farthest away from the heart, elasticity is not an issue. The structure of the varicose veins is different in various body parts. They are straight, curved, and sinusoidal in shape. A portion of such ascending sinusoidal vein is considered for this research work. Biocompatibility is the final issue to be resolved. The biomaterials have been used extensively for biomedical applications, and their behavior with the human body is called biocompatibility. There are various biomaterials which can be used instead of silver, like polymers (artificial and natural materials) and some metals [89].

### 5.3. Comparison of Results

The comparison between simulated values and experimental values has been presented in Table 4. All the values are in close agreement. The experimental values of flow rate and velocity are in between MATLAB and ANSYS simulated values.

In the MATLAB simulation, at 160 Reynolds number, the flow rate and velocity are 0.511 nL/s or 5.11 (0.1 nL/s), and 0.0455 m/s or 4.44 (cm/s), respectively. In the ANSYS Fluent simulation, at 161.3 Reynolds number, the flow rate and velocity are 0.6 nL/s or 6.0 (0.1 nL/s), and 0.06088 m/s, respectively. In the experiment, at 164.88 Reynolds number, the flow rate and velocity are 0.5843 nL/s or 5.843 (0.1 nL/s), and 0.05843 m/s or 5.843 (cm/s), respectively. The experimental and simulated results are found in close agreement for other Reynolds numbers, which are also used in this research. In the next section, a comparison of simulated and experimental values of this research work with the previous research of other microchannels, has been given. However, there is still no research data available on the flow rate and velocity measurement for ASMC and comparisons; that is the novelty of this research. The measurements of flow rate and velocity for other types of microchannels are available [24,68,90,91]. The deviation in the measurements have different reasons, like reduced or increased frictional factors, different types of microchannels, and a difference of applied pressure. All these factors can alter the measurements.

## 6. Conclusions

The purpose of this research was the simulations, fabrication, and testing of ascending sinusoidal microchannels. The research work includes computer simulations for blood flow rate and velocity using MATLAB FUZZY LOGIC and ANSYS Fluent. Furthermore, a bioengineered vein (silver based three curved ASMC) is fabricated to calculate the flow rate and velocity of human blood. Moreover, a comprehensive comparison has been presented between simulated and experimental values. Silver is a low cost and biocompatible material. Bioengineered veins can be implanted in the varicose (tortuous) veins that lead to better blood flow. These bioengineered veins can also be fabricated with other biocompatible materials, like polymers. These bioengineered veins can be made with any vein length, shape, and design. This is the novel solution of better blood flow through the ligated great saphenous vein. A bioengineered vein of similar length and diameter can also be fabricated and implanted in place of ligated saphenous veins. This research posed some limitations. Firstly, this channel was limited to a single-phase laminar flow regime, and neglected the venous leaflet valve. Secondly, the natural elasticity of the vein is neglected in this study, and third, is the frictional factor used in this research. In future, we will simulate a model that will consider all these limitations, in order to produce more physiological results.

## Figures and Tables

**Table 1:** Extensive details of different microchannels. PEFC: Polymer electrolyte fuel cell; WEDM: Wire electric discharge machining; PEM/GDL: Gas diffusion layer and GDL/GFC: Gas flow channel.

References	Length	Channel Type	Fabrication Technique	Fluid	Flow Rate	Simulation/Experiment	Applications
Abed et al. [7]	95 mm	Trapezoidal	Not reported	Water	Not reported	ANSYS Fluent 6.3	Heat exchangers
Shojaeian and Kosar [8]	1000 µm	Circular	Not reported	Water	Not reported	ANSYS Fluent 14.0	Heat transfer characteristics
Zhang et al. [37]	45 × 20 × 2 mm^3^	Straight rectangular	WEDM	Water	4.8 mL/min	Experiment	Heat transfer characteristics
Afzal and Kim [10]	2.8 mm	Straight and serpentine	Not reported	Blood	Not reported	ANSYS CFD	Flow dynamics and mixing behavior
Ashrafi et al. [11]	4 mm	Straight and serpentine	Not reported	Water	Not reported	Numerical	PEM fuel cells
Cardiel et al. [12]	2 cm	T-shaped	Standard soft lithography	Water	4000 μL/h	Not Reported	Instability behavior of membranes
Masuda et al. [63]	30 × 3.2 mm^2^	Straight	Simple machining process	Water	58,116 and 232 cm^3^/min	PEFC	Mass transportation PEM/GDL and GDL/GFC
Nishimura and Matsune [64]	14 mm	Sinusoidal	Simple machining process	Water	2.5 m^3^/s	Experiment	Heat transfer characteristics
Lu et al. [65]	183 mm	Sinusoidal	Simple machining process	Water/air	0.02–0.2 mL/min	Experiment	PEM fuel cell

**Table 2:** Ranges of membership functions.

Membership Functions	Reynolds Number		Pressure (kPa)		Curve Height (mm)		% Loss		Flow Rate (0.1 nL/s)		Velocity (cm/s)	
	Ranges	MFs	Ranges	MFs	Ranges	MFs	Ranges	MFs	Ranges	MFS	Ranges	MFs
MF1	0–500	SMALLEST	1–1.25	LOW	5–6	SMALL	0.1–0.55	SMALL	0–5	SMALL	2–4.5	SMALL
MF2	0–1000	SMALLER	1–1.5	MEDIUM	5–7	MEDIUM	0.1–1	MEDIUM	0–10	MEDIUM	2–7	MEDIUM
MF3	500–1000	SMAL	1.25–1.5	HIGH	6–7	HIGH	0.55–1	LARGE	5–10	HIGH	4.5–7	HIGH

**Table 3:** Comparison between Mamdani’s value and MATLAB simulation.

Category	Flow Rate (0.1 nL/s)	Velocity (cm/s)
Mamdani’s value	5.15	4.65
MATLAB simulation	5.11	4.55
Difference	0.04	0.1
Error percentage	0.78%	2.19%

**Table 4:** Comparison between simulated values and experimental values.

MATLAB Results	ANSYS Results	Experimental Results
Reynolds Number = 160	Reynolds Number = 161.33	Reynolds Number = 164.88
Flow Rate = 5.11 (0.1 nL/s)	Flow Rate = 6.0 (0.1 nL/s)	Flow Rate = 5.8 (0.1 nL/s)
Velocity = 4.55 (cm/s)	Velocity = 6.088 (cm/s)	Velocity = 5.8 (cm/s)
